# Prediction of therapeutic dropout in patients with addictions: Development and validation of the Predictors of Dropout from Addiction Treatment (PDAT) scale

**DOI:** 10.1371/journal.pone.0326853

**Published:** 2025-06-27

**Authors:** Carlos Miguel Sirvent-Ruiz, María Miranda, María de la Villa Moral-Jiménez

**Affiliations:** 1 Research and Teaching Department, Fundación Instituto Spiral, Madrid, Spain; 2 Department of Psychology, University of Oviedo, Oviedo, Spain; European University of Rome, ITALY

## Abstract

**Background:**

Withdrawal from addiction treatment is a frequent but difficult-to-predict contingency. We clarify and contextualize the concept of dropouts in addiction treatment, as well as the external and internal elements that most frequently lead to such dropouts. The main instruments used to measure dropout are summarized, after which a new tool, Predictors of Dropout from Addiction Treatment (PDAT) scale, is presented. The PDAT consists of four factors: 1) Motivation: desire to recover and to actively engage in current treatment; 2) Craving: longing for the use of substances and/or the substance addiction environment; 3) Problem awareness: level of insight, or degree of knowledge, and ability to objectify the problem and the disease, with the renunciations and limitations that this entails; and 4) Dysphoria: dyade inner restlessness – moodiness, i.e., emotional disturbance and depressive anticipation that precedes treatment withdrawal.

**Methods:**

The sample consisted of 243 addicted subjects in residential treatment, ranging in age from 18 to 63 years (average = 38.43, standard deviation = 10.95), who completed an initial 26-item PDAT questionnaire. The factor structure of the PDAT was determined by factor analysis. Mixed effects logistic regressions and receiver operating characteristics curve (ROC) analyses were applied to assess the predictive validity of the PDAT. Results: The 13-item PDAT showed adequate reliability and convergent and discriminant validity, with both the general scale and each of its factors having predictive validity 7 and 15 days after administration.

**Conclusion:**

The scale is a useful instrument with proven clinical efficacy and brevity of application. In addition, its four factors are useful for targeting interventions based on the unbalanced factors.

## Introduction

The concept of “treatment dropout” (also referred to as attrition, premature termination, early treatment withdrawal or premature discontinuation) is a complex construct that has a wide range of definitions [1]. A joint interpretation is to consider it as a unilateral and premature interruption of a treatment before the necessary level of improvement or completion of therapeutic goals has been achieved [2–4]. However, the operationalization of this concept differs across studies, and several methods have been used for dropout identification: therapist judgment [5], nonattendance at a given number of sessions [6], failure to resume therapy after the initial appointment [7] or after the last agreed appointment [8], or a score on an outcome assessment or on a symptom reduction inventory [4].

For the purposes of this study and for the operationalization of the research, we defined dropout as the early withdrawal of treatment at the participant’s request and against the criteria of the therapeutic team [4,9,10], but we understand that there are many nuances to this.

Several metanalyses have reported dropout rates from psychosocial treatment for substance use disorder [2,11,12], with average values ranging from 19.7% [2] to 47% [12]. However, there has been a wide variability depending on the type of population treated, the substance studied, and the treatment characteristics [11]. Treatment dropout has been associated with an increased risk of poor outcome and relapse [13], defined in the Medical Subjects Headings thesaurus as the recurrence of a sign, symptom or disease (addiction in this case) after remission [14]. On the other hand, both retention (duration of uninterrupted participation in treatment, generally operationalized as the time from admission to discharge from treatment) and adherence (attendance at scheduled appointments and taking the medication indicated during treatment) [15] have been positively related to favourable outcomes [16,17]. However, it has been suggested that these two indicators should be accompanied by other indicators reflecting clinical status, well-being and quality of life indicators [16].

Among the predictors of dropout, craving deserves special mention as a construct that encompasses multiple meanings, including need to consume, anticipatory anxiety, and depressive or withdrawal symptoms [18,19]. The central role of craving as a predictor of addiction treatment outcomes needs to be further supported by research [20,21], but several studies have identified craving and substance use desire as a major cause of treatment dropout [22–25] and relapse [26–29]. However, Rohsenow et al. [30] found that craving did not predict treatment dropout but did predict the amount of cocaine consumed during the first three months posttreatment. Similarly, Wray et al. [31] found inconsistent relationships between craving and smoking relapse. Nevertheless, when craving is associated with other factors, such as increased stress and decreased mood, the likelihood of dropout is significantly increased [32,33].

In addition to craving, stress, anxiety sensitivity and low distress tolerance appear to be incremental and prospective predictors of treatment dropout [34,35], whereas high levels of motivation and perceived need for treatment are well-studied factors reducing the risk of drop-outs [36–38]. Other predictors of dropout include depression [39,40], younger age [41], cocaine use, criminal activity/incarceration and negative attitudes [42], physical distress (naltrexone treatment) [43], and personality disorders [44], especially histrionic and antisocial disorders [45], among others. Gender differences are slightly in favour of women [46]. It is worth noting the difference between patients and therapists in the reported reasons for drop-out. While both clinicians and patients indicated that staff connection problems was a main issue, clinicians also considered client motivation to be one of the main reasons, whereas patients indicated that lack of social support was a main additional reason [47].

Failure to follow a treatment program is not always attributable to the patient. Different studies place the responsibility for better retention on the therapeutic alliance, and specifically on the therapist’s competence and skills [48]. Dissatisfaction with the treatment and/or with unmet social needs are considered by patients as barriers to retention [49]. Poor clinical insight can also be an obstacle to treatment success [36] since better patient insight and clinical insight in general improve abstinence [50]. In this regard, treatment readiness may be an interesting predictor of engagement and retention [51]. Other authors [52] differentiate between efficacy and retention, noting that the latter can be predicted by both client and therapist characteristics, whereas efficacy in outpatient substance abuse treatment depends more on client characteristics and previous substance use. There also seems to be an effect of the substance used, with cocaine users having the highest drop out rates [42]. Although patients with concomitant mental disorders (dual disorder) have been the subject of multiple studies [53], ADHD was the only mental disorder significantly related to dropout. Rigorous reviews [54] found no significant differences between patients with co-occurring mental illness and addiction and the control group.

### Instruments for assessing the risk of dropout in addiction treatment

Of the instruments related to the assessment of dropout risk or related concepts (e.g., retention and adherence) in addiction treatment, only the *Recovery Attitude and Treatment Evaluator* (RAATE-Q1) and the *Recovery Attitude and Treatment Evaluator-Clinical Evaluation* (RAATE-CE) [55] were specifically designed for this purpose, with the RAATE-CE being directed to the therapist and not to the patient. Both RAATE-Q1 and RAATE-CE explore intrinsic aspects such as the patient’s medical and mental conditions as well as their attitude towards treatment and continuing care, while also measuring circumstances that are external to the patient (external support) and hence are not susceptible to modification [3].

### Justification of the need for this questionnaire and study aims

To our knowledge, there is no instrument that simultaneously measures the four most important elements of treatment failure: lack of motivation for treatment, craving, lack of awareness of the problem, and dysphoria or mood disorders. Therefore, the present study aims to develop and evaluate the psychometric properties of a brief self-report scale designed to predict the risk of dropout from addiction treatment, focusing on the four aforementioned intrinsic patient factors, and intended to be applicable at any point in treatment (early and late dropout).

## Materials and methods

### Participants and setting

Out of 2,804 subjects admitted to the Fundación Instituto Spiral (hereafter FIS) care centres within the 15-year period under consideration, 243 participated in the study. Only those who had completed at least one PDAT and had given their consent participated. Participants had been diagnosed with disorders due to substance use (either alcohol, other drugs, or both) and/or addictive behaviours (gambling disorder) according to the ICD-11 classification [56], and had received treatment for chemical and/or behavioural addictions at any time during the study period in the FIS care centres in Madrid, the Principality of Asturias or Castile Leon (all in Spain). The data were accessed on 13 March 2024 for research purposes. In total, participants responded to 878 PDAT questionnaires throughout the study period. Participants were in a residential treatment consisting on an integrative program with cognitive-behavioral psychotherapeutic techniques, sociotherapy, and pharmacotherapy, when necessary.

The participants consisted of 33.20% women and 66.80% men aged between 18 and 63 years (M = 38.43; SD = 10.95). Most of the participants (56.45%) had a medium socio-economic level, with 0.96% of the participants having a high level and 6.70% having a very low or destitute level. The level of education completed was lower secondary education for 37.45% of the participants, upper secondary education for 29.63%, while 10.70% were postgraduate and 3.70% had not completed primary education (socio-demographic data categories are in accordance with the criteria standardised by the regional funding institutions). Age at use initiation ranged from 10 to 44 years (M = 17.33; SD = 4.87). The primary reasons for admission were dependence on alcohol (30.86% of participants), cocaine (18.11%), cannabis (10.70%), cocaine plus alcohol (9.88%), opiates (6.58%), psychotropic drugs (4. 53%), opiates plus cocaine (4.12%), amphetamines and/or synthetic drugs (4.12%), cocaine plus cannabis (3.70%), polydrug use (3.2%), alcohol plus cannabis (2.06%), and pathologic gambling (2.06%). In terms of psychiatric comorbidity diagnosed according to ICD-11, 56.79% of the participants had comorbid disorders, of which 27.54% had personality disorders and related traits, 20.29% had mood disorders, 10.87% had schizophrenia and other primary psychotic disorders, 4.35% had neurodevelopmental disorders, 2.90% had disorders specifically associated with stress, 2.17% had feeding and eating disorders, 0.7% had anxiety and fear-related disorders, and 31.16% had more than one of the mentioned comorbid disorders.

### Instrument

Participants were administered the PDAT scale (in Spanish) on at least one occasion during their treatment. The mean number of PDATs administered per participant was 3.60. The timing of the first administration of the scale covered a wide time range from the day after admission to 1309 days after admission. The PDAT scale assesses patients’ risk of dropping out of treatment, and its four subscales describe the following vulnerability factors: Motivation, Craving, Problem awareness, and Dysphoria. Motivation refers to the desire to recover and to actively engage in current treatment. Craving represents the longing for substances and/or the substance addiction environment. Problem awareness corresponds to the level of insight or degree of knowledge and ability to objectify the problem and the disease, with the renunciations and limitations that this entails. Dysphoria refers to the dyade inner restlessness – moodiness, i.e., emotional disturbance and depressive anticipation that could precede treatment withdrawal.

The development of the PDAT scale involved, firstly, the construction of an item bank, for which an exhaustive literature review was carried out on the factors of dropout and/or adherence to addiction treatment identified in previous studies. In addition, a retrospective study with 46 patients who had voluntarily left treatment in FIS centres between 2007 and 2009 served, through an open protocol, to identify the most frequent causes of dropout and the factors that contribute to adherence to treatment from the perspective of both the patient and the therapist [57]. This resulted in an initial pool of 150 items assessing motivation, awareness of the problem, dysphoria, cravings and perception of treatment. The content of those items was evaluated by FIS psychologists and psychiatrists specialised in addiction treatment and subjected to a screening process until an initial refined version of 26 item of the scale was obtained. Those 26 self-reported items were assessed using a 5-point Likert scale, with scores ranging from 1-“Very Much in Agreement” to 5 -”Strongly Disagree” (or with equivalent response options: from 1-‘I feel vital/motivated/excited’ to 5-’I feel devitalised/unmotivated/disillusioned’), where some of the items were reverse-scored. Higher PDAT scores are expected to be associated with a greater risk of treatment dropout.

### Study design

An ex post-facto design was applied. A multivariate correlational design was used to evaluate the psychometric properties of a self-report measure that quantifies predictors of treatment dropout in addictions (PDAT) and to investigate the validity of this scale to predict dropout in addiction treatment.

### Procedure

The data were collected by professionals from the FIS therapeutic programme as part of routine patient care and were treated with the strictest confidentiality (including assignment of an identification code). The authors did not have access to information that could identify individual participants during or after the study. Patients were informed of the aims of the study and were asked to participate on a voluntary basis. Written informed consent was obtained from all participants. The study was conducted in accordance with the ethical provisions laid down in the 1964 Declaration of Helsinki and its subsequent amendments. The study was approved by the Ethics Committees of the Principality of Asturias and the Palencia Health Area (reference numbers CEImPA 2022.480 and CEIm 2022/033, respectively).

### Data analyses

The structure of the PDAT variables was examined using exploratory factor analysis and then a confirmatory factor analysis was performed to evaluate the structure obtained. Both analyses were performed on the first PDAT administered to each of the 243 participants (S2 Dataset and S3 Table). Confirmatory factor analysis was conducted on the same data that was used for the exploratory factor analysis, since this has been deemed suitable when testing the fit of the exploratory model under more restrictive specifications [58]. No data splitting was applied, as this strategy has been found to result in less accurate parameter estimates and high rates of non-convergence compared to retaining the entire sample across models, due to the reduction in sample size that occurs when the data are split [59]. Given the ordinal nature of the variables and their uni- and multivariate non-normality, the exploratory factor analysis was based on the polychoric correlation matrix. The adequacy of the sample was checked using the determination index, the Kaiser–Meyer–Olkin (KMO) index, and Bartlett’s test of sphericity. The number of factors to be retained was determined via parallel analysis, the estimation method used was the minimal residual method, and oblique rotation was applied using the Promax method. Items with factor loadings less than 0.6 and those that were isolated into factors with less than 3 items were progressively eliminated. For the confirmatory factor analysis, the diagonally weighted least squares estimator was used and items with an R^2^ of less than 0.4 were removed. Model fit was checked using the values of the comparative fit index (CFI), the Tucker–Lewis index (TLI), root mean square error of approximation (RMSEA) and standardized root mean square residual (SRMR).

The internal consistencies of the scale and its factors were examined by calculating the ordinal alpha reliability coefficient based on the polychoric correlation matrix [60]. The convergent validity of the measurement model was assessed by the average variance extracted (AVE) and the composite reliability (CR). Discriminant validity was assessed using the heterotrait-monotrait relationship of correlations (HTMT) between subscales.

Measure invariance of the factorial structure of the PDAT applied at different treatment times was checked by multigroup confirmatory factor analyses using polychoric correlations and the diagonally weighted least squares estimator. For this, the participants were divided into two groups: (a) participants who completed the PDAT during the first 30 days of treatment (142 participants, 58%) and (b) participants who completed the PDAT after the first 30 days of treatment (101 participants, 42%). Responses of 4 and 5 were collapsed by giving them both a score of 4 to avoid some items not containing all response options. Three levels of invariance were considered [61]: (1) configural invariance, equivalence of factor structure between groups; (2) metric invariance, equivalence of factor loadings between groups; and (3) scalar invariance, which requires item intercepts or thresholds to be equal between groups. We considered more restricted models to fit the data better when the CFI difference between more and less constraint nested models was equal or less than 0.01 [62,63].

Finally, the validity of the PDAT and each of its factors to predict treatment dropout or the intention to drop out 7 and 15 days after administration was evaluated using mixed effects logistic regressions. The response variable in these models was dropout or intention to drop out, specified as a dichotomous variable (yes = drop out or intention to drop out within 7/15 days of scale administration; no = continuation of treatment with no intention to drop out). The fixed effect structure included the average answer score calculated across all included items, time in treatment at the time of PDAT administration (in days), both added as fixed continuous predictors, as well the their two-way interaction. Participant ID code was specified as a random effect to account for lack of independence between responses from the same participant. Separate models were run for the general scale, including all 13 items, as well as for each of its factors, only including the four items corresponding to that specific factor. Dropout was defined as voluntary discharge by the patient before being recommended by the clinical staff, without a justified reason, and provided that the patient’s initial intention was to comply with a comprehensive program. As for the intention to drop out, an anti-drop out protocol is implemented as soon as the subject requests voluntary discharge. The measures of this protocol include, among others, the request to be discharged with a 48 hours’ notice. Two indicators of intention to drop out were defined: 1) predischarge request of a 48-hour reflection period, and 2) introduction of anti-craving drugs within 7 or 15 days of PDAT, even if the participant did not ultimately drop out. These drugs are administered only to subjects that show an intention to drop out, even if there is no registered discharge request and are, therefore, clear indicators of the intention to quit.

In addition to the mixed effects logistic regressions, we evaluated the predictive validity of the PDAT by testing if the area under the ROC curve (AUC) of the mixed effects logistic regressions was 0.5, which would indicate no discriminatory ability. We repeated this analyses for both for the general scale and each of the factors, and for both 7 and 15 days after administration.

Significance threshold was set at 0.05 for all analyses. Exploratory factor analysis was performed in JASP version 0.18.3 [64]. All other analyses were performed in R version 4.3.1 [65] (S4 Appendix). Confirmatory factor analysis was performed using the packages lavaan [66], semTools [67] and semPlot [68], mixed effects logistic regressions were performed using the package lme4 [69], and analyses based on the receiver operating characteristics curve was performed using pROC [70].

## Results

### Sample adequacy and factor structure of the PDAT scale

Sample adequacy and model fit were appropriate according to the determination index (matrix determinant = 0.0001), the KMO value (0.836), Bartlett’s test of sphericity (*χ*^2^ = 2110.748, df = 78, p < 0.001), CFI = 0.959, and TLI = 0.899.

The exploratory factor analysis yielded a structure of 13 items in four factors, which explained 69.81% of the variance. This scale will be referred to as PDAT-13 from here on (S1 Appendix). The four factors obtained assess Motivation, Craving, Problem awareness and Dysphoria. Four items initially considered to reflect the perception of treatment were not grouped under their own factor, but were instead initially designated to the Problem awareness factor (“This treatment seems right for me”) or the Dysphoria factor (“How do you receive the pressure of treatment? “, “This treatment overwhelms me” and “I find it difficult to carry out this treatment”), and were finally eliminated due to their low loadings. The remaining items loaded on the expected factor: four of them on the Motivation factor explaining 22.72% of the common variance, while three items loaded on each of the remaining three factors explaining 18.65% (Craving), 14.80% (Problem awareness), and 13.65% (Dysphoria) of the common variance. The factor structure and factor loadings for the PDAT-13 are shown in Table 1. All the factors had positive pairwise correlation coefficients greater than 0.3 except for Craving and Problem Awareness (0.123) (Table 2).

**Table 1:** Factor Structure of the PDAT-13 Estimated from an Exploratory Factor Analysis on the Initial 26 Item PDAT Administered to the 243 Participants.

Factor	Items	Factor loadings
**Motivation**	1- In terms of vitality, how do you feel?	0.800
	4- Regarding the desire to do things, how do you feel?	0.743
	7- Do you feel excited?	0.921
	9- I want to recover	0.782
**Craving**	3- I have imaginations or fantasies of using substances	0.907
	6- I have a desire to use substances	0.938
	10- When I have not been using substances regularly for a long time, I start to think about using them and I cannot get it out of my mind	0.806
**Problem awareness**	2- Honestly, I plan to stay in treatment for as long as I set myself, even if the team thinks otherwise	0.696
	11- I would leave treatment because I am able to continue on my own	0.786
	13- It is possible that I may leave treatment in the short term by my own choice	0.790
**Dysphoria**	5- Honestly, my attention is more on the outside than on the inside of the treatment.	0.707
	8- I feel anxiety, inner restlessness	0.647
	12- I’m feeling grumpy, upset	0.724

**Table 2:** Correlations between the Factors Extracted for PDAT-13.

Factor	Motivation	Craving	Problem awareness
**Motivation**			
**Craving**	0.426		
**Problem awareness**	0.347	0.123	
**Dysphoria**	0.639	0.468	0.437

### Analysis of the measurement model

**Fig 1:**
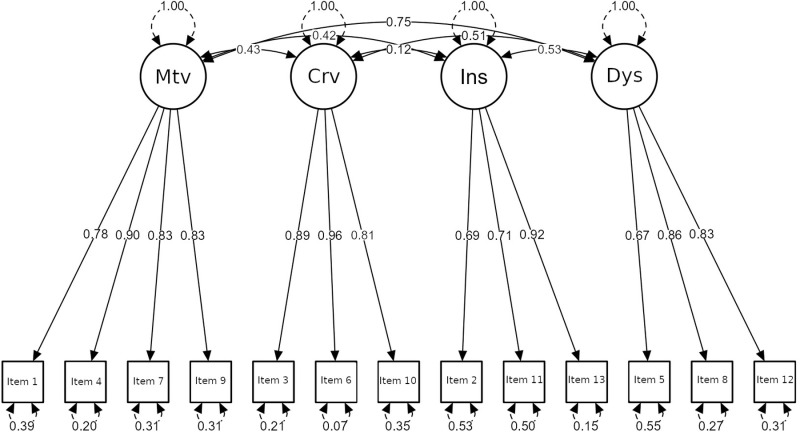
Estimated standardised parameters for the PDAT-13 measurement model. The items are represented in squares, and the factors are represented in circles: Mtv = Motivation; Crv = Craving; Ins = Insight; Dys = Dysphoria.

The fit of he confirmatory factor model estimated on the structure yielded by the exploratory factor analysis was adequate (CFI = 0.998, TLI = 0.997, RMSEA = 0.037, SRMR = 0.056, χ2 = 78.034, df = 59, p = 0.049) (Fig 1). All items loaded on their corresponding factors with significant factor loadings (p < 0.001).

### Internal consistency and convergent and discriminant validity of the PDAT-13

The PDAT-13 showed good internal consistency with an ordinal alpha of 0.889, ranging from 0.819 for the Problem awareness factor to 0.916 for the Craving factor. CR was 0.862 for Motivation, 0.891 for Craving, 0.780 for Problem awareness, and 0.803 for Dysphoria.

Regarding convergent validity, the AVE was 0.701 for Motivation, 0.789 for Craving, 0.609 for Problem Awareness, and 0.623 for Dysphoria. In terms of discriminant validity, the HTMT values between factors were adequate (Table 3).

**Table 3:** Heterotrait-Monotrait Relationship of Correlations for PDAT-13 Factors.

Factor	Motivation	Craving	Problem awareness
**Motivation**			
**Craving**	0.404		
**Problem awareness**	0.341	0.093	
**Dysphoria**	0.694	0.472	0.494

All correlations between the item scores were statistically significant except most of those between the items of the Craving and the Problem awareness factors, as well as the correlation between items 1 (Motivation) and 2 (Problem awareness) (Table 4).

**Table 4:** Polychoric Correlations between PDAT-13 Item Scores.

Item	1	2	3	4	5	6	7	8	9	10	11	12
**1**												
**2**	0.097											
**3**	0.257^***^	−0.009										
**4**	0.747^***^	0.178^**^	0.354^***^									
**5**	0.363^***^	0.388^***^	0.265^***^	0.411^***^								
**6**	0.313^***^	0.053	0.859^***^	0.427^***^	0.318^***^							
**7**	0.678^***^	0.273^***^	0.237^***^	0.696^***^	0.350^***^	0.268^***^						
**8**	0.524^***^	0.254^***^	0.408^***^	0.644^***^	0.551^***^	0.435^***^	0.586^***^					
**9**	0.581^***^	0.249^***^	0.360^***^	0.648^***^	0.343^***^	0.441^***^	0.746^***^	0.527^***^				
**10**	0.302^***^	0.092	0.729^***^	0.327^***^	0.273^***^	0.762^***^	0.221^***^	0.379^***^	0.322^***^			
**11**	0.129^*^	0.548^***^	0.029	0.184^**^	0.365^***^	0.123	0.205^**^	0.213^**^	0.345^***^	0.205^**^		
**12**	0.424^***^	0.272^***^	0.359^***^	0.602^***^	0.596^***^	0.410^***^	0.496^***^	0.699^***^	0.491^***^	0.336^***^	0.266^***^	
**13**	0.234^***^	0.617^***^	0.056	0.319^***^	0.341^***^	0.127	0.426^***^	.0327^***^	0.484^***^	0.088	0.638^***^	0.460^***^

* *p* < 0.05, ** *p* < 0.01, *** *p* < 0.001.

### Measurement invariance of the PDAT-13

Measurement invariance was observed between participants who completed the PDAT within 30 days of admission and participants who completed the PDAT after 30 days of treatment, for all levels considered (configural-metric levels: *χ*^*2*^ difference = 9.112, p* *= 0.427, CFI scaled difference = −0.002; metric-scalar levels: *χ*^*2*^ difference = 10.138, p* *= 0.985, CFI scaled difference = 0.000) (Table 5).

**Table 5:** Tests of Measurement Invariance of the PDAT-13 between Participants who Completed the PDAT within 30 Days of Admission (n = 142) and Participants who Completed the PDAT after 30 Days of Treatment (n = 101).

Model	*χ*2 (df)	CFI	TLI	RMSEA	SRMR	Robust *χ*2 difference	*χ*2 df difference	p (> *χ*2)
**Configural**	113.481 (118)	0.979	0.973	0.069	0.071			
**Metric**	124.494 (127)	0.982	0.978	0.062	0.071	9.112	9	0.427
**Scalar**	128.253 (149)	0.982	0.981	0.057	0.071	10.138	22	0.985

### Predictive validity of the PDAT-13 and its factors

The analysis of the predictive validity of the PDAT-13 was based on scores on 878 PDAT questionnaires that had been administered to the 243 participants throughout their treatment. The probability of dropping out or of having an intention to drop out within 7 and 15 days after PDAT administration increased significantly with increasing scores on both the general scale and on each of its four factors (Table 6). However, there were no significant interaction effects between the scores and the days in treatment at the time of PDAT administration (PDAT-13 7 days: β = 0.591, p = 0.196; PDAT-13 15 days: β = 0.391, p = 0.315; Motivation 7 days: β = 0.450, p = 0.186; Motivation 15 days: β = 0.278, p = 0.372; Craving 7 days: β = 0.405, p = 0.415; Craving 15 days: β = 0.446, p = 0.275; Problem awareness 7 days: β = 0.163, p = 0.719; Problem awareness 15 days: β = 0.073, p = 0.853; Dysphoria 7 days: β = 0.176, p = 0.713; Dysphoria 15 days: β = −0.009, p = 0.982).

**Table 6:** Odds Ratio, β Coefficient, Associated Standard Error (SE) and P-Values (p) of Mixed Effects Logistic Regressions and Absolute Difference between the 7-day Model and the 15-day Model Slopes.

Variable	Days since PDAT-13	*OR*	*β*	*SE*	*p*	Absolute difference between slopes
**PDAT-13**	7	2.6991	0.9929	0.2102	< 0.0001	0.1071
	15	2.4249	0.8858	0.1763	<0.0001	
**Motivation**	7	2.1278	0.7551	0.1694	< 0.0001	0.0967
	15	1.9316	0.6584	0.1459	< 0.0001	
**Craving**	7	1.7018	0.5317	0.2183	0.0149	0.0411
	15	1.6332	0.4906	0.1817	0.0069	
**Problem awareness**	7	1.8673	0.6245	0.1930	0.0012	0.0604
	15	1.7579	0.5641	0.1673	0.0007	
**Dysphoria**	7	2.0742	0.7296	0.2129	0.0006	0.0566
	15	1.9601	0.6730	0.1799	0.0002	

The slope of the 7-day model run for each factor was in all cases higher than that of the 15-day model, with the largest absolute difference found for the Motivation factor (Table 6).

**Fig 2:**
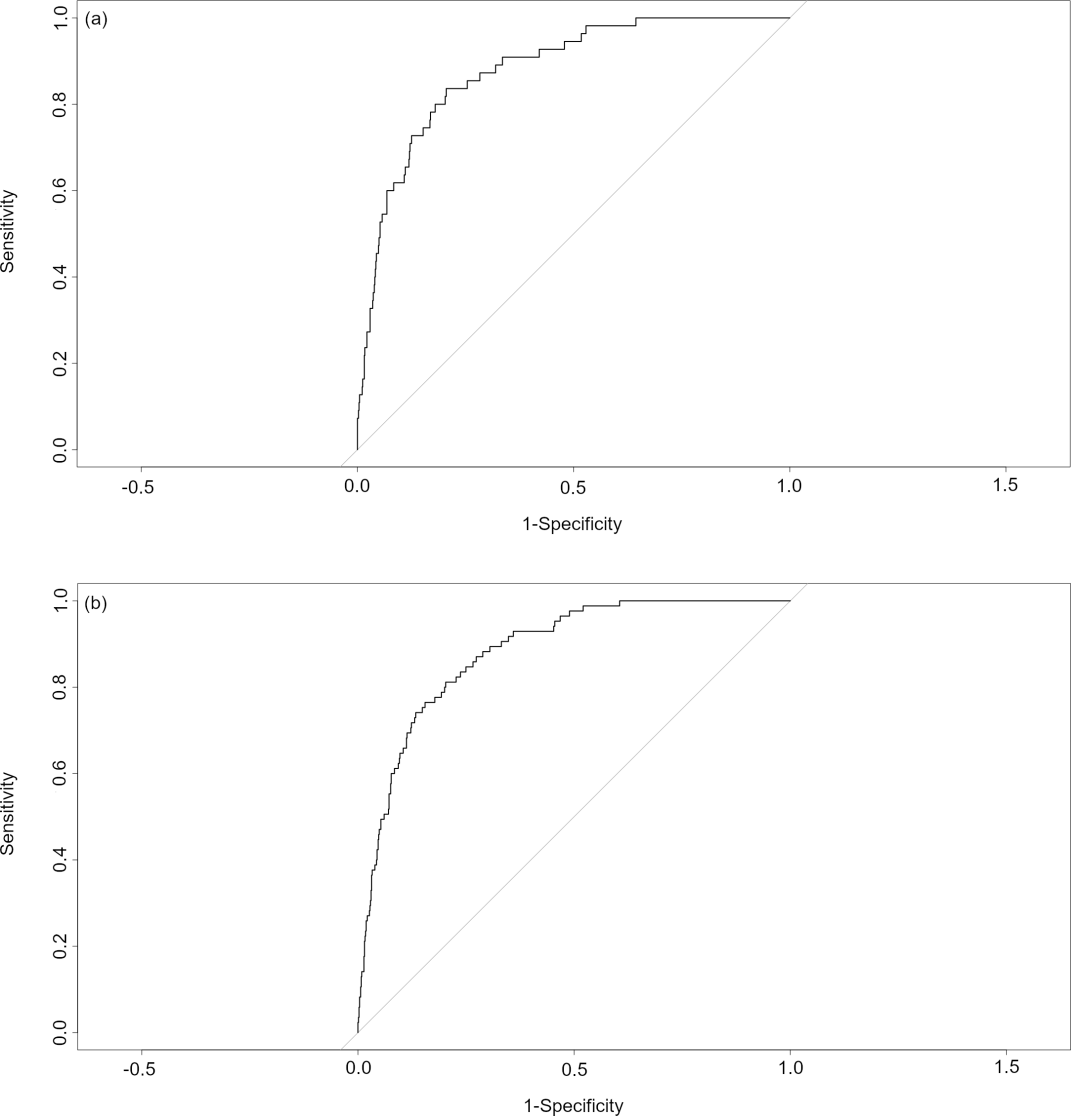
ROC Curves for the Mixed Effects Logistic Regressions of the PDAT-13 and each of its Factors. (a) 7 and (b) 15 days after PDAT-13 administration.

The AUC values were over 0.8 for the ROC curves for the mixed effects logistic regression models of the PDAT-13 and for each of its factors both in the 7 and 15 days after administration (Fig 2), and they were significantly different from 0.5 (Table 7).

**Table 7:** AUC, 95% Confidence Intervals for the AUC (95% CI), and AUC P-Values for the ROC Curves for the Mixed Effects Logistic Regressions of the PDAT-13 and each of its Factors in the a) 7 and b) 15 Days after Administration.

Variable	Days since PDAT-13	AUC	95% CI	p-value
**PDAT-13**	7	0.8797	0.8374-0.9219	< 0.001
	15	0.8835	0.8512-0.9159	< 0.001
**Motivation**	7	0.9007	0.8641-0.9373	< 0.001
	15	0.9037	0.8766-0.9309	< 0.001
**Craving**	7	0.9352	0.9049-0.9656	< 0.001
	15	0.9134	0.8839-0.9428	< 0.001
**Problem awareness**	7	0.9054	0.8636-0.9471	< 0.001
	15	0.9065	0.8758-0.9373	< 0.001
**Dysphoria**	7	0.8757	0.8324-0.919	< 0.001
	15	0.8840	0.8520-0.9161	< 0.001

## Discussion

The completion of addiction treatments has been associated with more favourable outcomes [17,71]. Therefore, early detection of variables that predict treatment dropout is key to increase treatment effectiveness [72]. The self-report scale developed here is brief, applicable both on admission and in later stages of treatment and has good psychometric properties. This scale consists of four factors that predict treatment dropout in the short term, which allows for the identification of subjects requiring early intervention, making it highly applicable in clinical and research settings.

Scale development focused exclusively on dynamic predictor variables intrinsic to the patient and to the interaction between the patient and their treatment process [24,41], all of which are amenable to monitoring, evaluation and eventual intervention [3]. Fixed or enduring external pre-treatment circumstances, such as socioeconomic and demographic variables, history and severity of use, and previous treatment, have previously been the most widely studied predictors of dropout [41]. This is probably because they are often routinely collected at the time of admission. Such fixed characteristics, however, have not shown consistent effects on the risk of dropping out [41,73], and several analyses have shown that the dynamic characteristics of the individual and its interaction with treatment are of greater importance as determinants of retention in addiction treatment [74,75]. Our results support this, as both the PDAT and each of its factors showed predictive validity for short-term dropout (at 7 and 15 days after administration).

From the initial 26-item PDAT scale, factor analysis revealed a 13-item solution on four dimensions (Motivation, Craving, Problem Awareness and Dysphoria). In line with previous studies [71,75–77], treatment perception was initially hypothesized to be a PDAT factor. However, items referring to this factor were assigned to the Problem awareness and Dysphoria factors and were eventually removed from the analysis due to their low factor loadings. This may be have been because participants who completed the PDAT in the initial phases of treatment had not been significantly affected by the restrictions and renunciations associated with the treatment process [78].

The domains of dropout assessed by the PDAT-13 partly coincide with those considered by the RAATE scales. However, the PDAT-13 explicitly assesses Dysphoria, which is not covered by the RAATE, although distress tolerance, a closely related concept, has been found to interact with external circumstances, motivation and willingness to treat in predicting dropout [37].

The pairwise correlations between the different factors of the PDAT-13 were adequate, except for the correlation between the Problem awareness factor and the Craving factors (Table 2), which was less than 0.2. The polychoric correlation between most of the items of these two factors was also low. This could be explained by a possible mediating effect of self-deception in addicted patients on the relationship between these two latent constructs, where a high level of self-deception together with low problem awareness negatively impacts the subject’s ability to identify the level of craving [36,79,80], which in turn interferes with their adherence to treatment [3].

Previous studies have identified variations in predictors of dropout as a function of the patient’s stage of treatment [25,74]. There are also studies suggesting that patients who drop out early in treatment may differ from those who drop out in the longer term [17,35]. Therefore, in the future, it would be critical to determine whether the PDAT-13 factors differ in the relative influence of the time a patient has been in treatment on their respective contributions to how well the scale predicts retention. This knowledge would allow, in clinical practice, the development of optimal strategies aimed at preventing dropout specifically in each phase of addiction treatment. It would also be important to analyse the possible interaction effects between PDAT-13 factors on the risk of dropout, as well as the potential effect of demographic factors as moderators of the relationship between PDAT predictors and the likelihood of dropout.

### Limitations

Criterion validity has not been tested through correlational analyses of related constructs for either the PDAT-13 or each of its factors. However, predictive validity was observed after 7 and 15 days of administration both for the PDAT-13 scale and for each of its factors, specifically supporting the criterion validity of the PDAT-13 for the intended purpose of anticipating dropout. Additionally, it has not been possible to verify whether the PDAT has predictive validity in the medium term after its administration. Perhaps the main drawback in this sense is that, in the centres where the scale has been applied, there is an intervention protocol for patients at risk of dropping out, which limits such predictive ability. However, the fact that that protocol is triggered after an alert issued to a subject due to a high score on the scale reinforces the predictive value of the scale presented here.

## Conclusions

We suggest that PDAT-13 is a novel and clinically useful scale for assessing the risk of dropping out from treatment for addictions based on dynamic characteristics of the individual and their interaction with treatment. The scale has good psychometric properties, and both the PDAT-13 and each of its four factors showed predictive validity for dropout in the days following administration. Therefore, this new scale allows both the identification of patients at risk of dropping out and of their most deficient factors, which guides the therapist in adapting interventions early on and facilitates additional follow-up and support. Although there are numerous instruments that assess different aspects of addiction, there are only a limited number that assess the process of therapeutic dropout/retention, and there is none that simultaneously registers the four most important elements of therapeutic failure (lack of motivation for treatment, craving, lack of awareness of the problem, and dysphoria or mood disorders). Hence there ought to be an interest in a brief instrument that is as complete as possible, quick to apply, effective in its results and, that serves as an indication to the therapist of the specific area of potential dropout on which they have to intervene to prevent dropout. However, we highlight that it would be interesting to be able to determine how the different PDAT-13 factors behave in terms of the risk of dropout in the different phases of treatment. We have doubts about predictions in the medium or even long term, as the instrument is configured to predict dropout in the short term. It is worth noting that the treatment itself is supposed to neutralize withdrawal factors.

## Supporting information

S1 Appendix Questionnaire. PDAT-13 items.
(DOCX)

S2 Dataset Study data. (XLSX)

S3 Table Item correspondence. Correspondences between the PDAT-13 and PDAT-26 item numbers.
(XLSX)

S4 Appendix Code. R code used for confirmatory factor analysis, mixed effects logistic regressions and ROC curves.
(DOCX)
