# Supplementary material for: Prediction of therapeutic dropout in patients with addictions: Development and validation of the Predictors of Dropout from Addiction Treatment (PDAT) scale
Source: PLoS One. 2025 Jun 27;20(6):e0326853. doi: 10.1371/journal.pone.0326853 (PMC12204563; doi:10.1371/journal.pone.0326853)
Supplement: S1 Appendix — PDAT-13 items. (DOCX) [file pone.0326853.s001.docx]

S1. Questionnaire

PDAT-13 Items

The response options are scored from 1 to 5 in the order in which they appear below (direct scoring) for items 1, 4, 7 and 9, while items 2, 3, 5, 6, 8, 10, 11, 12 and 13 are scored from 5 to 1 (inverse scoring).

**1. In terms of vitality, how do you feel?**

I feel vital
 I feel more vital than devitalised
 I feel neither vital nor devitalised
 I feel more devitalised than vital
 I feel devitalised

**2. Honestly, I plan to stay in treatment for as long as I set myself, even if the team thinks otherwise**

Very much in agreement
 Agree
 Neither agree nor disagree
 Disagree
 Strongly Disagree

**3. I have imaginations or fantasies of using substances**

Very much in agreement
 Agree
 Neither agree nor disagree
 Disagree
 Strongly Disagree

**4. Regarding the desire to do things, how do you feel?**

I feel motivated, I feel like doing things.
I feel more motivated than unmotivated
I feel neither motivated nor unmotivated
I feel more unmotivated than motivated
I feel unmotivated, with no desire to do things

**5. Honestly, my attention is more on the outside than on the inside of the treatment**

Very much in agreement
 Agree
 Neither agree nor disagree
 Disagree
 Strongly Disagree

**6. I have a desire to use substances**

Very much in agreement
 Agree
 Neither agree nor disagree
 Disagree
 Strongly Disagree

**7. Do you feel excited?**

I feel excited
 I feel more excited than disillusioned
 I feel neither excited nor disillusioned
 I feel more disillusioned than excited
 I feel disillusioned

**8. I feel anxiety, inner restlessness**

Very much in agreement
 Agree
 Neither agree nor disagree
 Disagree
 Strongly Disagree

**9. I want to recover**

Very much in agreement
 Agree
 Neither agree nor disagree
 Disagree
 Strongly Disagree

**10. When I have not been using substances regularly for a long time, I start to think about using them and I cannot get it out of my mind**

Very much in agreement
 Agree
 Neither agree nor disagree
 Disagree
 Strongly Disagree

**11. I would leave treatment because I am able to continue on my own**

Very much in agreement
 Agree
 Neither agree nor disagree
 Disagree
 Strongly Disagree

**12- I'm feeling grumpy, upset**

Very much in agreement
 Agree
 Neither agree nor disagree
 Disagree
 Strongly Disagree

**13- It is possible that I may leave treatment in the short term by my own choice**

Very much in agreement
 Agree
 Neither agree nor disagree
 Disagree
 Strongly Disagree
