# Supplementary material for: Prediction of therapeutic dropout in patients with addictions: Development and validation of the Predictors of Dropout from Addiction Treatment (PDAT) scale
Source: PLoS One. 2025 Jun 27;20(6):e0326853. doi: 10.1371/journal.pone.0326853 (PMC12204563; doi:10.1371/journal.pone.0326853)
Supplement: S4 Appendix — R code used for confirmatory factor analysis, mixed effects logistic regressions and ROC curves. (DOCX) [file pone.0326853.s004.docx]

**S4. Code**

**R code used for confirmatory factor analysis, mixed effects logistic regressions and ROC curves**

**a) R code for confirmatory factor analysis of PDAT-13**

We used the data in file S2_Data. Only the first questionnaire of each patient was used in this analysis.

#Upload necessary packages

library(lavaan)

library(semTools)

#Specify model and run CFA

PDAT13.model.promax<-'Motivation =~ PDAT26_Item.2 + PDAT26_Item.10 + PDAT26_Item.17 + PDAT26_Item.20

+ Craving =~ PDAT26_Item.9 + PDAT26_Item.13 + PDAT26_Item.21

+ Dysphoria =~ PDAT26_Item.11 + PDAT26_Item.19 + PDAT26_Item.25

+ Problemawareness =~ PDAT26_Item.6 + PDAT26_Item.232+ PDAT26_Item.26'

CFA.PDAT13.promax<-cfa(PDAT13.model.promax,data=first.PDAT13,estimator='WLSMV',ordered=TRUE)

summary(CFA.PDAT13.promax, fit.measures=TRUE,standardized=TRUE)

#Get ordinal alpha, AVE, CR and HTMT:

reliability(CFA.PDAT13.promax,return.total=TRUE)

compRelSEM (CFA.PDAT13.promax)

htmt(CFA.PDAT13.promax, data=first.PDAT13, ordered=TRUE)

#Test for measurement invariance between two temporal groups

#Configural model

CFA.PDAT13.multigroup.config<-cfa(PDAT13.model.promax ,data=first.PDAT13.collapsed,ordered=TRUE,group="temporal.group")

#Metric model

CFA.PDAT13.multigroup.weak<-cfa(PDAT13.model.promax,data=first.PDAT13.collapsed,ordered=TRUE,group="temporal.group",group.equal=c("loadings"))

#Scalar model

CFA.PDAT13.multigroup.strong<-cfa(PDAT13.model.promax,data=first.PDAT13.collapsed,ordered=TRUE,group="temporal.group",group.equal=c("loadings","intercepts"))

#Model comparison

lavTestLRT(CFA.PDAT13.multigroup.config,CFA.PDAT13.multigroup.weak, CFA.PDAT13.multigroup.strong)

fitMeasures(CFA.PDAT13.multigroup.config, c("cfi","cfi.scaled")) - fitMeasures(CFA.PDAT13.multigroup.weak, c("cfi","cfi.scaled"))

fitMeasures(CFA.PDAT13.multigroup.weak, c("cfi","cfi.scaled")) - fitMeasures(CFA.PDAT13.multigroup.strong, c("cfi","cfi.scaled"))

**b) R code for mixed effects logistic regressions to test predictive validity of PDAT13 and its subscales**

We used all cases in file S2 Data.

"dropout.48h.anticraving.7days" is a dichotomous variable that takes the value 1 in those cases in which drop out, 48h reflection period request, or administration of anticraving medication occurred within 7 days of PDAT administration.

"dropout.48h.anticraving.15days" is a dichotomous variable that takes the value 1 in those cases in which drop out, 48h reflection period request, or administration of anticraving medication occurred within 15 days of PDAT administration.

The data provided in S2 Data allow the use of any other time interval.

#Upload necessary packages

library(lme4)

#Run glms to test the validity of the general PDAT-13 scale for predicting the risk of dropping out in the 7 and 15 days after administration

glmer.PDAT13.7days<-glmer(dropout.48h.anticraving.7days~PDAT13.scores+(1|Patient.ID),data=S2_Data,family="binomial")

summary(glmer.PDAT13.7days)

glmer.PDAT13.15days<-glmer(dropout.48h.anticraving.15days~PDAT13.scores+(1|Patient.ID),data=S2_Data,family="binomial")

summary(glmer.PDAT13.15days)

#The variable PDAT13.scores was substituted by each of the subscales scores (Motivation.scores, Craving.scores, Problem.awareness.scores or Dysphoria.scores) to determine the validity of each of them to predict drop out risk.

**c) R code for receiver operating characteristic (ROC) curve analyses to assess predictive validity of PDAT13 and its subscales**

We used all cases in file S2 Data.

#Upload necessary packages

library(pROC)

#Assign the values predicted by the mixed effects logistic regressions to a variable in the data table

prob.PDAT13.7d=predict(glmer.PDAT13.7days,type=c("response"))

prob.PDAT13.15d=predict(glmer.PDAT13.15days,type=c("response"))

S2_Data$prob.PDAT13.7d = prob.PDAT13.7d

S2_Data$ prob.PDAT13.15d = prob.PDAT13.15d

#Plot the ROC curves and calculate the AUC area for the predicted probability values of the general PDAT-13 scale to assess the risk of dropping out in the 7 and 15 days after administration

roc.PDAT13.7d <- roc(dropout.48h.anticraving.7days~prob.PDAT13.7d, data=S2_Data, ci=TRUE)

roc.PDAT13.7d

plot(roc.PDAT13.7d, legacy.axes = TRUE)

roc.PDAT13.15d <- roc(dropout.48h.anticraving.15days~prob.PDAT13.15d, data=S2_Data)

roc.PDAT13.15d

plot(roc.PDAT13.15d, legacy.axes = TRUE)

#The fit "glmer.PDAT13.7days" was substituted by each of the subscales fits (glmer.MOTIVATION.7days, glmer.CRAVING.7days, etc) to assess the validity of each of the subscales to predict drop out risk.

# Calculate the p-value for the test of the null hypothesis that the area under the ROC curve is 0.5, i.e. PDAT-13 has no predictive ability.

(v.roc.PDAT13.7d<-var(roc.PDAT13.7d))

b.roc.PDAT13.7d<-roc.PDAT13.7d$auc - .5

(se.roc.PDAT13.7d<-sqrt(v.roc.PDAT13.7d))

z.roc.PDAT13.7d<-(b.roc.PDAT13.7d/se.roc.PDAT13.7d)

2*pt(-abs(z.roc.PDAT13.7d),df=Inf)

## #The curve "roc.PDAT13.7d" was replaced by each of the calculated ROC curves to evaluate each of them separately.
